# Using electronic health record system triggers to target delivery of a patient-centered intervention to improve venous thromboembolism prevention for hospitalized patients: Is there a differential effect by race?

**DOI:** 10.1371/journal.pone.0227339

**Published:** 2020-01-16

**Authors:** Oluwafemi P. Owodunni, Elliott R. Haut, Dauryne L. Shaffer, Deborah B. Hobson, Jiangxia Wang, Gayane Yenokyan, Peggy S. Kraus, Jonathan K. Aboagye, Katherine L. Florecki, Kristen L. W. Webster, Christine G. Holzmueller, Michael B. Streiff, Brandyn D. Lau

**Affiliations:** 1 Division of Acute Care Surgery, Department of Surgery, The Johns Hopkins University School of Medicine, Baltimore, Maryland, United States of America; 2 Department of Anesthesiology and Critical Care Medicine, The Johns Hopkins University School of Medicine, Baltimore, Maryland, United States of America; 3 Department of Emergency Medicine, The Johns Hopkins University School of Medicine, Baltimore, Maryland, United States of America; 4 Armstrong Institute for Patient Safety and Quality, Johns Hopkins Medicine, Baltimore, Maryland, United States of America; 5 Department of Health Policy and Management, The Johns Hopkins Bloomberg School of Public Health, Baltimore, Maryland, United States of America; 6 Department of Nursing, The Johns Hopkins Hospital, Baltimore, Maryland, United States of America; 7 Department of Biostatistics, Johns Hopkins Bloomberg School of Public Health, Baltimore, Maryland, United States of America; 8 Department of Pharmacy, The Johns Hopkins Hospital, Baltimore, Maryland, United States of America; 9 Division of Hematology, Department of Medicine, The Johns Hopkins Hospital, Baltimore, Maryland, United States of America; 10 Russell H. Morgan Department of Radiology and Radiological Science, The Johns Hopkins Hospital, Baltimore, Maryland, United States of America; 11 Division of Health Sciences Informatics, The Johns Hopkins Hospital, Baltimore, Maryland, United States of America; Universite de Bretagne Occidentale, FRANCE

## Abstract

**Background:**

Racial disparities are common in healthcare. Venous thromboembolism (VTE) is a leading cause of preventable harm, and disparities observed in prevention practices. We examined the impact of a patient-centered VTE education bundle on the non-administration of preventive prophylaxis by race.

**Methods:**

A post-hoc, subset analysis (stratified by race) of a larger nonrandomized trial. Pre-post comparisons analysis were conducted on 16 inpatient units; study periods were October 2014 through March 2015 (baseline) and April through December 2015 (post-intervention). Patients on 4 intervention units received the patient-centered, nurse educator-led intervention if the electronic health record alerted a non-administered dose of VTE prophylaxis. Patients on 12 control units received no intervention. We compared the conditional odds of non-administered doses of VTE prophylaxis when patient refusal was a reason for non-administration, stratified by race.

**Results:**

Of 272 patient interventions, 123 (45.2%) were white, 126 (46.3%) were black, and 23 (8.5%) were other races. A significant reduction was observed in the odds of non-administration of prophylaxis on intervention units compared to control units among patients who were black (OR 0.61; 95% CI, 0.46–0.81, *p*<0.001), white (OR 0.57; 95% CI, 0.44–0.75, *p*<0.001), and other races (OR 0.50; 95% CI, 0.29–0.88, *p* = 0.015).

**Conclusion:**

Our finding suggests that the patient education materials, developed collaboratively with a diverse group of patients, improved patient’s understanding and the importance of VTE prevention through prophylaxis. Quality improvement interventions should examine any differential effects by patient characteristics to ensure disparities are addressed and all patients experience the same benefits.

## Introduction

Health disparities occur across many dimensions and result from a range of social determinants.[1] Racial disparities are common among hospitalized patients, which limits the impact of quality improvement strategies for all patient populations, potentially resulting in health disparities and undue costs.[2,3] Large nationally representative studies demonstrate differences in health care and outcomes among white, black, and other minority races.[4] Patient information in electronic health record (EHR) systems allows an analysis of health-related outcomes by different socioeconomic strata and racial/ethnic groups.[2,3] Health disparities are multifaceted and may be the result of inequitable (meaning unjust) care, inequalities in socioeconomic, behavioral or other factors. Health equity stands on the principle that each person’s full health potential is not restrained by any form of social injustice or inequality. Conversely, health care inequality connotes variability and disproportionate healthcare access and utilization and stems from the concept that access to healthcare should be the same for everyone.[5,6] A systematic examination of inequalities proposes nine modern theories to explain the differences in health for different groups. [7] Some authors point to specific factors such as institutional or large-scale societal biases (e.g., rural vs. urban location, academic vs. non-academic hospital), and other factors such as provider factors (e.g., age, sex, race/ethnicity), whether explicit or implicit bias, are integral contributors to existing disparities.[1,8]

Venous thromboembolism (VTE) is a leading cause of preventable in-hospital morbidity and mortality.[9,10] Consequently, universal risk assessment and prescription of risk-appropriate prophylaxis for VTE is recommended for all hospitalized patients.[11] Efforts have improved provider compliance with prescribing risk-appropriate VTE prophylaxis and increased patient acceptance of doses.[12,13] Yet some disparities in VTE development are due to the genetic makeup of different races.[4,14–16] However, socioeconomic risk factors also play a role.[17] There are no differences in recommended prophylaxis regimens by race, even though evidence suggests that rates of VTE vary among race/ethnic groups.[18,19] Over a decade, a multidisciplinary VTE Collaborative implemented a variety of interventions to optimize the prescription and delivery of risk-appropriate VTE prophylaxis.[20] They demonstrated that implementation of a computer clinical decision support tool eliminated race and sex-based disparities in VTE prophylaxis prescription,[21,22] and was an unintended consequence, or “halo effect,”[23] of applying quality improvement strategies to optimize care for all patients.

While these interventions improved prescription of risk-appropriate VTE prophylaxis,[21,24,25] they discovered that prescribed doses were not reliably administered to patients,[26,27] contributing to the development of potentially preventable VTE events.[28,29] Furthermore, they discovered that administration of VTE prophylaxis differed significantly by race; the leading cause of non-administration was patient refusal.[27] To reduce the frequency of non-administered doses and potentially address this issue in patients of all races, this group involved a racially diverse group of patients in developing a patient education bundle about VTE and prevention and published the handout in 8 languages.[27,30] The education was designed to improve health literacy as part of a strategy to improve patient-centered care and health promotion.[30] The bundle is coupled with a real-time alert built into the EHR system to identify and target hospitalized patients who miss doses and has been associated with decreases in non-administration.[10] Observation from studies that have looked at the outcomes of medical innovations and inequalities indicates that before the beneficial effects of the implemented change there is usually a disproportionate effect observed in different groups.[31,32] We hypothesized that use of this education bundle would provide equal care to all patients regardless of race, and concomitantly reduce disparities in the administration of VTE prophylaxis. The purpose of this study was to examine the impact of the patient-centered VTE education bundle on the non-administration of preventive prophylaxis by race.

## Methods

### Study setting and design

This project is a post-hoc, subset analysis (stratified by race) of a larger prospective study that examined the overall impact of the bundle on improving administration of prescribed pharmacologic VTE prophylaxis for hospitalized patients, which has been previously described.[33] The current analysis used a controlled pretest-posttest parallel experimental design to evaluate the impact of the bundle on prophylaxis administration by race; study period was April 1, 2015, to December 31, 2015.[10] The study included 16 adult medical and surgical nursing units, excluding intensive care, at The Johns Hopkins Hospital. Using a convenience sample, we assigned 4 units to receive the intervention: 2 surgical and 2 medical. The remaining 12 units (6 surgical and 7 medical) served as controls. io:dx.doi.org/10.17504/protocols.io.9u3h6yn.

We performed a power calculation and, based on the number of patients and the very large number of doses historically prescribed, found we would have sufficient power for this study.[26] Our blinded biostatistician team (JW, GY) was not involved in the outcome determination and analyses were conducted from June 2016 through November 2017 and followed the TREND guidelines for nonrandomized controlled trials.[34]

### Intervention

The intervention included a real-time alert, triggered when a patient missed a dose of their pharmacologic VTE prophylaxis, and patient education. The alert was built into our hospital EHR system and used the unit name field to identify participating units. Our trained health educator carried a pager and the EHR system paged and e-mailed only the educator with the name and unit location of the patient whom missed the prophylaxis dose. The health educator then visited the floor to engage the bedside nurse and determine the cause of the missed dose. If the patient had refused the dose, the patient was provided with the education bundle. If the dose was missed for other reasons, the educator explained the importance of prophylaxis for VTE prevention to the documenting nurse and tried to resolve it. No intervention was provided to patients and nurses on control units. Although the health educator was not consistently present in the hospital, all missed doses required documentation by the bedside nurse as administered or nonadministered, and the reason for nonadministration.

### Patient education bundle

Patients could choose to receive one or more components from the patient education bundle. The education bundle included: 1) a one-on-one dialogue with the nurse educator, 2) a 2-page, paper handout (in English or one of seven other languages), and 3) a 10-minute video (bit.ly/bloodclots) viewed on a handheld tablet. We developed this education bundle using a modified Delphi method to build consensus on the content and modes of delivery of VTE prevention information to hospitalized patients.[30] We had input from over 400 stakeholders from three national organizations and our local hospital Patient and Family Advisory Council. A detailed description of the education bundle is published elsewhere.[10,30,35]

### Statistical analysis

Our primary outcome of interest was the proportion of doses missed due to patient refusal or for other documented reasons (holds on orders for a procedure, patient away from bed) stratified by racial group. Thus, we reported patient visits (admissions/encounters) rather than unique patients.[10] We compared the change in the rates of VTE prophylaxis administration for all included patient visits before the intervention (October 1, 2014 –March 31, 2015) to after the intervention (April 1 –December 31, 2015).[10] We also hypothesized a differential effect on medicine and surgery units and thus, performed a pre-specified stratified analysis by floor type. Patient-level and nurse-level demographic characteristics for the pre-intervention period were delineated by arm (Table 1). Two-sample t-tests with equal variance were used to compare age. Chi-square tests were used to compare gender, race and floor type. The non-parametric Wilcoxon rank-sum tests were used to compare the number of dosages and length of stay.

**Table 1:** Demographic characteristics of patient visits by race in the intervention and control groups.

	Intervention pre-period				Intervention post-period				Control pre-period				Control post-period			
	White	Black	Others	p	White	Black	Others	p	White	Black	Others	p	White	Black	Others	p
**Unique Visits**	1,088	941	193		1,533	1308	270		2,850	2307	500		4,432	3469	761	
**Unique Patients**	922	762	170		1,279	1039	221		2,428	1860	431		3,669	2635	648	
**Unique Nurses**	121	100	27		131	114	29		409	268	67		421	335	79	
**Mean Age (SD), years**^a^	56.1 (17.3)	51.8 (17.4)	51.7 (16.4)	< .001	57.7 (16.6)	51.8 (18.1)	50.6 (17.6)	< .001	58.5 (16.7)	54.3 (16.7)	53.3 (16.7)	< .001	58.3 (16.5)	53.2 (16.8)	53.5 (17.6)	< .001
**Sex, n (%)**^b^				0.97				0.10				< .001				< .001
**Male**	526 (48.3%)	449 (47.8%)	93 (48.2%)		738 (89.1%)	637 (48.7%)	149 (55.2%)		1599 (56.1%)	1083 (46.9%)	288 (57.6%)		2438 (55.0%)	1712 (49.4%)	435 (57.2%)	
**Female**	562 (51.7%)	491 (52.2%)	100 (51.8%)		795 (51.9%)	671 (51.3%)	121 (44.8%)		1251 (43.9%)	1224 (53.1%)	212 (42.4%)		1994 (45.0%)	1755 (50.6%)	328 (42.8%)	
**Floor Type, n (%)**^b^				< .001				< .001				< .001				< .001
**Surgery Floors**	717 (65.9%)	319 (33.9%)	119 (61.7%)		1012 (66.0%)	482 (36.9%)	153 (56.7%)		1732 (60.8%)	528 (22.9%)	270 (54.0%)		2736 (61.7%)	907 (26.1%)	433 (56.9%)	
**Medicine Floors**	371 (34.1%)	622 (66.1%)	74 (38.3%)		521 (34.0%)	826 (63.1%)	117 (43.3%)		1118 (39.2%)	1779 (77.1%)	230 (46.0%)		1696 (38.3%)	2562 (73.9%)	328 (43.1%)	
**Median Number of Prescribed Doses per Patient visit (Q1, Q3)**	7 (3–14)	6 (3–11)	6 (3–14)	< .001	8 (4–14)	6 (3–12)	6.5 (3–13)	< .001	8 (4–15)	7 (3–13)	8 (4–14)	< .001	8 (4–15)	7 (3–14)	8 (4–15)	< .001
**Mean (SD)**^c^	10.5 (11.3)	8.5 (8.6)	11.3 (14.0)		11.2 (11.9)	9.72 (15.5)	10.4 (11.0)		12.0 (14.1)	10.3 (10.9)	10.8 (10.9)		11.7 (12.1)	10.8 (12.5)	12.7 (17.9)	
**Median Length of Stay, days (Q1-Q3)**	4 (2–7)	3 (2–6)	4 (2–8)	< .001	5 (3–8)	4 (2–7)	4 (2–7)	< .001	5 (2–8)	4 (2–7)	5 (2–8)	< .001	5 (3–8)	4 (2–8)	5 (2–9)	< .001
**Mean (SD)**^c^	6.0 (5.8)	5.2 (6.4)	7.6 (11.4)		6.5 (6.7)	6.0 (9.3)	6.3 (7.9)		7.3 (9.4)	6.0 (8.0)	7.2 (8.8)		7.5 (10.4)	6.5 (8.7)	8.3 (12.4)	

^a^ The p values were calculated using two-sample t-tests with equal variances.

^b^ The p values were calculated using chi-square tests.

^c^ The p values were calculated using Wilcoxon rank-sum tests.

For race, specialty, and time comparisons we used generalized linear mixed-effects models to account for correlation within floor and nurse, and multiple outputations to account for multiple VTE doses per patient across nurses or units. This method selected one VTE prophylaxis dose per patient and reiterated the procedure 1000 times to bootstrap the *P* values and corresponding 95% confidence intervals (CI) for the comparisons.[36]

The models included group (intervention vs. control) and time (pre- vs. post-intervention) stratified by race as the primary predictors. To estimate the conditional odds ratios (OR) and 95% CI, we used the binomial family with the logit link command and the Poisson family with the log link for the conditional proportions. Stratified analyses were performed by unit type (medicine and surgery) using the same models.

All comparisons were performed at < 0.05 level of statistical significance. We performed manual medical record chart review to determine missing patient sex. Statistical analyses were performed using Stata version 14.1 MP—Parallel Edition (College Station, Texas 77845). The primary study “Education Bundle to Decrease Patient Refusal of VTE Prophylaxis” ClinicalTrials.gov NCT02402881.

### Study approval

The research application was approved by the Johns Hopkins University Institutional Review Board. The requirement for written informed consent was waived for participants before inclusion in this study. However, participants provided verbal consent to receive the education and the intervention was administered if they were willing to be engaged by the nurse educator. Patient-level data was analyzed after approval by the IRB for its use without consent.

## Results

Overall, 19 652 patient visits in which at least 1 dose of VTE prophylaxis medication was prescribed during their patient’s hospitalization were included. Table 1 (and S1 Table) delineate the clinical and demographic characteristics of patient visits by race. By race, the proportion of patients was similar for the intervention and control units and in the pre- and post-intervention periods (Table 1). Mean age was significantly different by arm and intervention period between race categories (*p*<0.001). Males accounted for a higher proportion of patients on control vs. intervention units. The median number of prescribed VTE doses per hospitalization was significantly different in the pre- and post-intervention periods on both control and intervention units (Table 1).

### Intervention delivery

Of 726 patient visits eligible for an intervention, 364 (50.1%) were white, 307 (42.3%) were black, and 55 (7.6%) were other races. Interventions were implemented with 272/726 (37.5%) unique patients (Table 2). The proportion of patients who received the intervention by race were, 123/364 (33.7%) white, 126/307 (41.0%) black, and 23/55 (41.8%) other races. Significantly more non-white patients received an intervention compared to white patients (41.0% vs. 33.7%, *p* = 0.040, respectively).

**Table 2:** Proportion of interventions delivered compared by floor type and race.

Variables	Total	White	Black	Other	Black+ Other	P Value^a^
**Total eligible for intervention, n**	726	364	307	55	362	0.040
**Total received intervention, n (%)**	272 (37.5%)	123 (33.7%)	126 (41.0%)	23 (41.8%)	149 (41.0%)	
**Surgery**						
**Total eligible for intervention, n**	289	171	97	21	118	0.022
**Total received intervention, n(%)**	66 (22.8%)	31 (18.1%)	29 (29.9%)	6 (28.6%)	35 (30.0%)	
**Medicine**						
**Total eligible for intervention, n**	437	193	210	34	244	0.844
**Total received intervention, n(%)**	206 (47.1%)	92 (47.7%)	97 (46.2%)	17 (50.0%)	114 (46.7%)	

^a^ The *p* value compares the proportion of interventions completed for eligible white patients versus non-white (black + other) patients

### VTE prophylaxis medication administration by race

**Fig 1:**
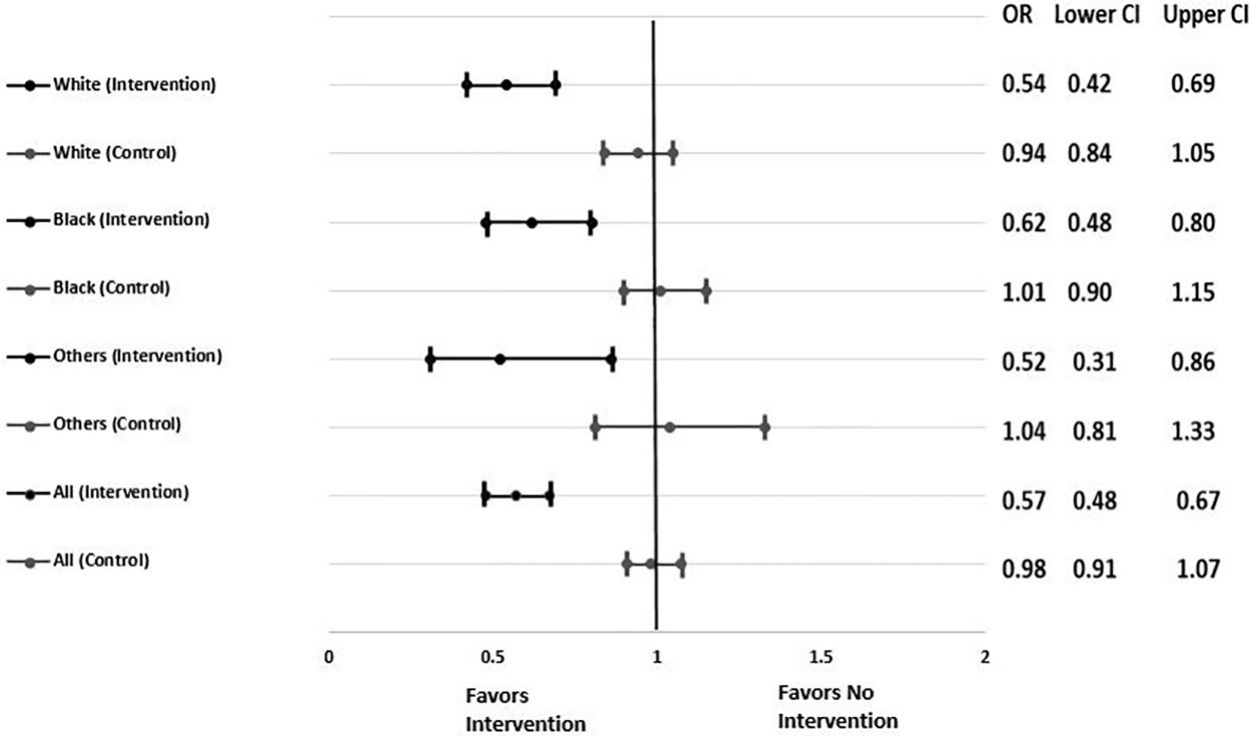
Effect of the patient-centered education bundle, comparing the conditional odds ratios for the intervention and control arms stratified by race. Fig 1 shows the effect of the patient-centered education bundle by comparing the conditional odds ratios for the intervention and control arms stratified by race. Odds ratios (OR) and 95% confidence intervals (CI) are reported.

The odds of nonadministration of VTE prophylaxis declined on intervention units by 38% (OR 0.62, 95% CI, 0.48 to 0.80) for black patients, by 46% (OR 0.54, 95% CI, 0.42 to 0.69) for white patients, and by 48% (OR 0.52, 95% CI 0.31 to 0.86) for other races (Fig 1). No change in odds of nonadministration of VTE prophylaxis was observed on control units for any race group. Upon testing the interaction between the odds of nonadministration on intervention units compared to control units, a significant decline was observed among patients who were black (OR 0.61; 95% CI, 0.46 to 0.81), white (OR 0.57; 95% CI, 0.44 to 0.75), and other races (OR 0.50; 95% CI, 0.29 to 0.88, Fig 1).

### Reason for VTE prophylaxis nonadministration by race

The odds of dose refusal on intervention units decreased pre-post by 43% (OR 0.57; 95% CI; 0.42 to 0.78) among black patients, by 48% (OR 0.52; 95% CI, 0.38 to 0.70) among white patients, and by 58% (OR 0.42; 95% CI, 0.21 to 0.82) among other races (Table 3). The odds of dose refusal remained unchanged on control units. The decline in refused doses on intervention units was statistically significantly different from control units for patients who were black (OR 0.57; 95%, 0.41 to 0.79), white (OR 0.55; 95%, 0.39 to 0.77), and other races (OR 0.41; 95%, CI 0.20 to 0.84). No significant differences were observed in the decline of non-administered doses for other reasons between intervention and control units (Table 3).

**Table 3:** Proportion of doses missed stratified by race for all reasons, refusal and other reasons: Comparisons between pre- vs. post-intervention by treatment group.

Race/Period		Intervention	Control	OR (95% CI) *p* value^a^
**Any Missed Dose**				
Black	Pre-Intervention % (95% CI)	7.4% (4.0%, 13.7%)	12.3% (8.6%, 17.5%)	0.57 (0.25, 1.28) *p* = 0.173
	Post-Intervention % (95% CI)	4.9% (2.6%, 9.0%)	12.3% (8.7%, 17.5%)	0.35 (0.15, 0.78) *p* = 0.011
	Odds Ratio Post/Pre (95% CI) *p value*^a^	0.62 (0.48, 0.80) *p*<0.001	1.01 (0.90, 1.15) *p* = 0.820	0.61 (0.46, 0.81) *p*<0.001^b^
White	Pre-Intervention % (95% CI)	10.6% (5.8%, 19.5%)	14.6% (10.3%, 20.8%)	0.69 (0.31, 1.54) *p* = 0.366
	Post-Intervention % (95% CI)	6.2% (3.4%, 11.5%)	13.9% (9.8%, 19.6%)	0.39 (0.18, 0.89) *p* = 0.024
	Odds Ratio Post/Pre (95% CI) *p value*^a^	0.54 (0.42, 0.69) *p*<0.001	0.94 (0.84, 1.05) *p* = 0.246	0.57 (0.44, 0.75) *p*<0.001^b^
Other	Pre-Intervention % (95% CI)	8.8% (4.4%, 17.4%)	13.9% (9.5%, 20.5%)	0.59 (0.25, 1.39) *p* = 0.229
	Post-Intervention % (95% CI)	4.9% (2.4%, 10.2%)	14.3% (9.8%, 20.8%)	0.30 (0.12, 0.74) *p* = 0.009
	Odds Ratio Post/Pre (95% CI) *p value*^a^	0.52 (0.31, 0.86) *p* = 0.011	1.04 (0.81, 1.33) *p* = 0.773	0.50 (0.29, 0.88) *p* = 0.015^b^
**Dose Refused by Patient**				
Black	Pre-Intervention % (95% CI)	4.9% (2.0%, 11.7%)	7.9% (4.8%, 13.1%)	0.59 (0.19, 1.80) *p* = 0.355
	Post-Intervention % (95% CI)	3.0% (1.2%, 7.2%)	8.0% (4.8%, 13.2%)	0.34 (0.11, 1.02) *p* = 0.055
	Odds Ratio Post/Pre (95% CI) *p value*^a^	0.57 (0.42, 0.78) *p*<0.001	1.01 (0.88, 1.17) *p* = 0.868	0.57 (0.41, 0.79) *p* = 0.001^b^
White	Pre-Intervention % (95% CI)	6.7% (2.8%, 16.0%)	9.3% (5.7%, 15.4%)	0.70 (0.23, 2.12) *p* = 0.527
	Post-Intervention % (95% CI)	3.7% (1.6%, 9.0%)	8.8% (5.4%, 14.5%)	0.38 (0.13, 1.17) *p* = 0.092
	Odds Ratio Post/Pre (95% CI)	0.52 (0.38, 0.70) *p*<0.001	0.94 (0.82, 1.08) *p* = 0.366	0.55 (0.39, 0.77) *p*<0.001^b^
Other	Pre-Intervention % (95% CI)	6.1% (2.4%, 15.8%)	8.6% (5.0%, 14.8%)	0.70 (0.22, 2.25) *p* = 0.547
	Post-Intervention % (95% CI)	2.8% (1.0%, 7.9%)	8.9% (5.2%, 15.1%)	0.28 (0.08, 0.98) *p* = 0.047
	Odds Ratio Post/Pre (95% CI) *p value*^a^	0.42 (0.21, 0.82) *p* = 0.011	1.03 (0.76, 1.40) *p* = 0.831	0.41 (0.20, 0.84) *p* = 0.015^b^
**Other Reason for Missed Dose (not patient refused)**				
Black	Pre-Intervention % (95% CI)	1.7% (1.0%, 2.8%)	2.9% (2.2%, 3.8%)	0.57 (0.33, 01.00) *p* = 0.052
	Post-Intervention % (95% CI)	1.5% (0.9%, 2.3%)	2.9% (2.3%, 3.8%)	0.49 (0.28, 0.84) *p* = 0.011
	Odds Ratio Post/Pre (95% CI) *p value*^a^	0.86 (0.58, 1.29) *p* = 0.464	1.01 (0.84, 1.23) *p* = 0.891	0.85 (0.55, 1.32) *p* = 0.465^b^
White	Pre-Intervention % (95% CI)	2.8% (1.8%, 4.4%)	3.8% (2.9%, 4.8%)	0.74 (0.43, 1.25) *p* = 0.255
	Post-Intervention % (95% CI)	1.8% (1.21%, 2.9%)	3.6% (2.8%, 4.6%)	0.50 (0.29, 0.86) *p* = 0.0134
	Odds Ratio Post/Pre (95% CI) *p value*^a^	0.64 (0.45, 0.91) *p* = 0.014	0.94 (0.81, 1.11) *p* = 0.478	0.68 (0.46, 1.01) *p* = 0.0584^b^
Other	Pre-Intervention % (95% CI)	0.9% (0.0%, -)	3.7% (2.6%, 5.23%)	0.40 (0.18, 0.90) *p* = 0.038
	Post-Intervention % (95% CI)	1.5% (0.7%, 3.2%)	3.8% (2.7%, 5.4%)	0.38 (0.17, 0.86) *p* = 0.0281
	Odds Ratio Post/Pre (95% CI) *p value*^a^	0.98 (0.39, 2.43) *p* = 0.964	1.04 (0.71, 1.51) *p* = 0.842	0.94 (0.36, 2.49) *p* = 0.905^b^

OR, odds ratio; CI, confidence interval.

^a^ P values for the odds ratios were calculated using multiple outputation of the generalized linear mixed-effects models with the binomial family and a logit link.

^b^ Two way interactions were performed including pre vs. post time period, and control vs. intervention units

### Stratified analysis by floor type (surgery and medicine) by race

The pre-specified analysis by floor type revealed a decline in the odds of nonadministration of VTE prophylaxis for all race groups (Table 4). On surgery intervention units, nonadministration significantly decreased pre-post among patients who were black (OR 0.53; 95% CI, 0.33 to 0.85), white (OR 0.57; 95% CI, 0.40 to 0.82), and other races (OR 0.30; 95% CI, 0.12 to 0.75). When comparing the odds of nonadministered doses on surgery intervention and control units, there was a significant difference for patients of other races (OR 0.33; 95% CI 0.12–0.91), but no significant difference for white or black groups.

**Table 4:** Subgroup analysis of floor type (surgery and medicine) effect on proportion of prescribed venous thromboembolism prophylaxis doses by race.

Surgery				
Period		Intervention	Control	*p* value^a^
**Any Missed Dose**				
Black	Pre-Intervention, % (95% CI)	5.2% (2.9%, 9.4%)	9.7% (6.9%, 13.7%)	0.50 (0.23, 1.06) p = 0.072
	Post-Intervention, % (95% CI)	2.9% (1.6%, 5.2%)	7.9% (5.6%, 11.0%)	0.34 (0.16, 0.71) p = 0.004
	Odds Ratio Post/Pre (95% CI) *p value*^a^	0.53 (0.33, 0.85) p = 0.008	0.78 (0.62, 0.99) p = 0.039	0.68 (0.41, 1.14) p = 0.145^b^
White	Pre-Intervention, % (95% CI)	4.8% (2.8%, 8.1%)	9.2% (6.7%, 12.6%)	0.48 (0.24, 0.97) p = 0.042
	Post-Intervention, % (95% CI)	2.8% (1.7%, 4.8%)	7.9% (5.8%, 10.8%)	0.33 (0.16, 0.67) p = 0.002
	Odds Ratio Post/Pre (95% CI) *p value*^a^	0.57 (0.40, 0.82) p = 0.002	0.84 (0.73, 0.98) p = 0.028	0.68 (0.46, 1.01) p = 0.054^b^
Other	Pre-Intervention, % (95% CI)	5.7% (3.1%, 10.3%)	9.2% (6.1%, 13.7%)	0.58 (0.27, 1.28) p = 0.178
	Post-Intervention, % (95% CI)	1.8% (0.7%, 4.9%)	8.3% (5.7%, 12.1%)	0.19 (0.06, 0.59) p = 0.004
	Odds Ratio Post/Pre (95% CI) *p value*^a^	0.30 (0.12, 0.75) p = 0.010	0.89 (0.62, 1.28) p = 0.541	0.33 (0.12, 0.91) p = 0.031^b^
**Patient Refused Dose**				
Black	Pre-Intervention, % (95% CI)	2.7% (1.2%, 5.9%)	4.7% (3.0%, 7.4%)	0.54 (0.20, 1.44) p = 0.219
	Post-Intervention, % (95% CI)	1.4% (0.6%, 3.1%)	3.9% (2.5%, 6.1%)	0.34 (0.12, 0.92) p = 0.033
	Odds Ratio Post/Pre (95% CI) *p value*^a^	0.50 (0.24, 1.03) p = 0.061	0.80 (0.59, 1.09) p = 0.162	0.62 (0.29, 1.35) p = 0.228^b^
White	Pre-Intervention, % (95% CI)	2.2% (1.1%, 4.4%)	4.4% (2.9%, 6.7%)	0.47 (0.19, 1.16) p = 0.100
	Post-Intervention, % (95% CI)	1.3% (0.6%, 2.8%)	3.5% (2.3%, 5.3%)	0.36 (0.14, 0.91) p = 0.031
	Odds Ratio Post/Pre (95% CI) *p value*^a^	0.60 (0.34, 1.06) p = 0.079	0.79 (0.64, 0.97) p = 0.024	0.77 (0.42, 1.41) p = 0.393^b^
Other	Pre-Intervention, % (95% CI)	3.8% (-, -)^c^	5.1% (3.0%, 8.7%)	0.72 (0.26, 1.96) p = 0.521
	Post-Intervention, % (95% CI)	0.8% (0.1%, 4.9%)	3.9% (2.4%, 6.5%)	0.19 (0.03, 1.31) p = 0.093
	Odds Ratio Post/Pre (95% CI) *p value*^a^	0.20 (0.03, 1.16) p = 0.072	0.74 (0.47, 1.19) p = 0.214	0.27 (0.04, 1.66) p = 0.158^b^
**Other Reason for Missed Dose (not patient refused***)*				
Black	Pre-Intervention, % (95% CI)	2.2% (1.1%, 4.5%)	4.0% (2.7%, 6.2%)	0.53 (0.23, 1.23) p = 0.140
	Post-Intervention, % (95% CI)	1.3% (0.0%, -0.0%)	3.2% (2.2%, 4.7%)	0.40 (0.19, 0.81) p = 0.011
	Odds Ratio Post/Pre (95% CI) *p value*^a^	0.59 (0.34, 1.01) p = 0.052	0.78 (0.55, 1.11) p = 0.167	0.75 (0.40, 1.42) p = 0.374^b^
White	Pre-Intervention, % (95% CI)	2.3% (1.3%, 4.2%)	3.9% (2.8%, 5.6%)	0.58 (0.28, 1.18) p = 0.133
	Post-Intervention, % (95% CI)	1.3% (0.7%, 2.4%)	3.6% (2.6%, 5.1%)	0.35 (0.17, 0.73) p = 0.005
	Odds Ratio Post/Pre (95% CI) *p value*^a^	0.56 (0.37, 0.84) p = 0.006	0.91 (0.76, 1.10) p = 0.334	0.62 (0.39, 0.98) p = 0.042^b^
Other	Pre-Intervention, % (95% CI)	0.2% (0-, -)^c^	3.1% (1.9%, 5.1%)	0.47 (0.11, 1.99) p = 0.307
	Post-Intervention, % (95% CI)	0.04% (0-, -)^c^	3.6% (2.3%, 5.6%)	0.32 (0.07, 1.54) p = 0.155
	Odds Ratio Post/Pre (95% CI) *p value*^a^	0.78 (0.14, 4.38) p = 0.781	1.15 (0.70, 1.89) p = 0.571	0.68 (0.12, 3.97) p = 0.667^b^
**Medicine**				
**Any Missed Dose**				
Black	Pre-Intervention, % (95% CI)	13.3% (8.0%, 22.1%)	16.6% (12.7%, 21.7%)	0.77 (0.39, 1.49) *p* = 0.432
	Post-Intervention, % (95% CI)	9.1% (5.5%, 15.2%)	17.4% (13.4%, 22.7%)	0.46 (0.24, 0.90) *p* = 0.022
	Odds Ratio Post/Pre (95% CI) *p value*^a^	0.65 (0.48, 0.88) *p* = 0.005	1.08 (0.93, 1.24) *p* = 0.314	0.60 (0.43, 0.84) *p* = 0.003^b^
White	Pre-Intervention, % (95% CI)	22.3% (13.4%, 37.2%)	20.6% (15.6%, 27.1%)	1.10 (0.56, 2.16) *p* = 0.781
	Post-Intervention, % (95% CI)	12.9% (7.7%, 21.6%)	20.9% (16.0%, 27.3%)	0.54 (0.28, 1.06) *p* = 0.072
	Odds Ratio Post/Pre (95% CI) *p value*^a^	0.51 (0.36, 0.70) *p*<0.001	1.03 (0.87, 1.21) *p* = 0.743	0.49 (0.34, 0.71) *p*<0.001^b^
Other	Pre-Intervention, % (95% CI)	14.4% (7.3%, 28.6%)	19.0% (13.3%, 27.1%)	0.71 (0.31, 1.64) *p* = 0.426
	Post-Intervention, % (95% CI)	10.5% (5.2%, 21.1%)	21.1% (15.2%, 29.4%)	0.42 (0.18, 0.99) *p* = 0.048
	Odds Ratio Post/Pre (95% CI) *p value*^a^	0.69 (0.33, 1.49) *p* = 0.321	1.16 (0.82, 1.64) *p* = 0.409	0.60 (0.27, 1.33) *p* = 0.207^b^
**Patient Refused Dose**				
Black	Pre-Intervention, % (95% CI)	11.6% (6.1%, 22.0%)	13.5% (9.6%, 19.0%)	0.84 (0.37, 1.89) *p* = 0.669
	Post-Intervention, % (95% CI)	7.2% (3.8%, 13.8%)	14.0% (10.0%, 19.5%)	0.47 (0.21, 1.06) *p* = 0.069
	Odds Ratio Post/Pre (95% CI) *p value*^a^	0.59 (0.42, 0.82) *p* = 0.002	1.05 (0.90, 1.23) *p* = 0.542	0.56 (0.39, 0.81) *p* = 0.002^b^
White	Pre-Intervention, % (95% CI)	18.2% (9.6%, 34.6%)	16.2% (11.4%, 22.9%)	1.14 (0.50, 2.60) *p* = 0.748
	Post-Intervention, % (95% CI)	9.8% (5.1%, 18.9%)	16.5% (11.7%, 23.1%)	0.53 (0.23, 1.22) *p* = 0.135
	Odds Ratio Post/Pre (95% CI) *p value*^a^	0.48 (0.33, 0.69) *p*<0.001	1.03 (0.86, 1.23) *p* = 0.772	0.47 (0.31, 0.70) *p*<0.001^b^
Other	Pre-Intervention, % (95% CI)	12.3% (5.4%, 2.8%)	13.6% (8.8%, 21.0%)	0.89 (0.33, 2.41) *p* = 0.813
	Post-Intervention, % (95% CI)	7.6% (3.3%, 17.8%)	15.9% (10.6%, 23.9%)	0.42 (0.15, 1.17) *p* = 0.098
	Odds Ratio Post/Pre (95% CI) *p value*^a^	0.58 (0.25, 1.34) *p* = 0.205	1.22 (0.81, 1.81) *p* = 0.340	0.48 (0.19, 1.20) *p* = 0.116^b^
**Other Reason for Missed Dose (not patient refused)**				
Black	Pre-Intervention, % (95% CI)	1.4% (0.7%, 2.5%)	2.4% (1.7%, 3.4%)	0.56 (0.27, 1.13) *p* = 0.104
	Post-Intervention, % (95% CI)	1.6% (0.8%, 3.1%)	2.8% (2.0%, 3.8%)	0.57 (0.27, 1.20) *p* = 0.139
	Odds Ratio Post/Pre (95% CI) *p value*^a^	1.17 (0.68, 2.02) *p* = 0.571	1.15 (0.91, 1.46) *p* = 0.204	1.02 (0.57, 1.80) *p* = 0.958^b^
White	Pre-Intervention, % (95% CI)	3.5% (1.9%, 6.6%)	3.6% (2.6%, 5.2%)	0.96 (0.46, 2.02) *p* = 0.914
	Post-Intervention, % (95% CI)	2.7% (1.4%, 5.2%)	3.7% (2.6%, 5.1%)	0.72 (0.33, 1.56) *p* = 0.399
	Odds Ratio Post/Pre (95% CI) *p value*^a^	0.75 (0.42, 1.37) *p* = 0.352	1.01 (0.76, 1.34) *p* = 0.940	0.75 (0.38, 1.46) *p* = 0.393^b^
Other	Pre-Intervention, % (95% CI)	0.02% (0-, -)^c^	4.5% (2.7%, 7.4%)	0.50 (0.09, 2.89) *p* = 0.439
	Post-Intervention, % (95% CI)	2.3% (0.7%, 7.2%)	4.3% (2.6%, 7.0%)	0.52 (0.15, 1.83) *p* = 0.305
	Odds Ratio Post/Pre (95% CI) *p value*^a^	1.00 (0.16, 6.08) *p* = 1.000	0.979 (0.54, 1.76) *p* = 0.920	1.03 (0.17, 6.35) *p* = 0.974^b^

OR, odds ratio; CI, confidence interval.

^a^ P values for the odds ratios were calculated using multiple outputation of the generalized linear mixed-effects models with the binomial family and a logit link.

^b^ Two way interactions were performed including pre vs. post time period, and control vs. intervention units for Surgery

^c^ Not provided by models, due to small numbers

On medicine intervention units, nonadministered doses significantly decreased pre-post among patients who were black (OR 0.65; 95% CI, 0.48 to 0.88) and white (OR 0.51; 95% CI, 0.36 to 0.70; Table 4). No significant difference was observed among patients of other races (OR 0.69 95% CI 0.33–1.49). When comparing the odds of nonadministration on medicine intervention and control units, there was a significant decline among patients who were black (OR 0.60; 95% CI, 0.43 to 0.84) and white (OR 0.49; 95% CI, 0.34 to 0.71), but not other races (OR 0.60; 95% CI, 0.27 to 1.33). The proportion of refused doses decreased significantly for both white and black patients on medical units.

## Discussion

In this study, we conducted a post-hoc analysis by race to determine the impact of a patient-centered education bundle on disparities related to the administration of prescribed doses of VTE prophylaxis. We found that the intervention was equitable and effective for all patients regardless of race. The odds of a patient refusing a prophylaxis dose significantly decreased on intervention units for all race groups after the education bundle was implemented, with approximately the same effect size. We hypothesized a differential effect between medicine and surgery units because of the heterogeneity in service types. However, the odds of nonadministered prophylaxis doses significantly declined for both clinical services. These findings suggest that the patient education materials, which were developed collaboratively with a diverse group of patients, improved the patient’s understanding of VTE and the importance of prevention through prophylaxis. This concept of health literacy may positively impact the quality of care for all patients and the potential to reduce disparities in health outcomes.

Our patient education bundle included an interactive conversation between the nurse educator and patient and was most likely associated with rate declines in missed VTE prophylaxis doses regardless of patient race. One likely reason was the patient-centered, inclusive nature of the education. We originally translated the handout into 8 languages (Arabic, Chinese, English, Korean, Nepalese, Portuguese, Russian and Spanish) to ensure we reached the racial distribution of patients at The Johns Hopkins Hospital. Some patients responded favorably when given material in their native language. For the patient education video, we consciously recruited patients from a variety of racial backgrounds, and of varying ages and sexes (bit.ly/bloodclots). Another likely reason for rate declines was the interactive approach we used, which is consistent with prior research of health literacy.[21,30,35] Health literacy depends on effective communication in which information is explained clearly to make the topic easily understood. When we surveyed patients and families before developing the bundle, face-to-face conversations with a health care provider was the preferred method of receiving information about VTE.[30] Our patient-centered approach ensured that patients participated in their care pathway and made well-informed decisions. Thus, we demonstrated that real-time delivery of a patient-centered education intervention can improve health literacy and concomitantly reduce patient refusal and non-administration of pharmacologic VTE prophylaxis. It is important to note that providers must seek out patient preferences for receiving education.[37]

Despite the remarkable progress observed, there remained missed doses of VTE prophylaxis for reasons other than patient refusal. The available data does not allow additional exploration to determine the reason for this trend. However, we speculate that nursing factors, unit culture, or variation in practice relative to contraindications could have influenced decision-making; our intervention may have indirectly affected these issues. The direct effect of our intervention demonstrates that the bundle was not restricted to any specific patient populations and can be implemented regardless of clinical service. Similar declines in the odds of non-administration on both medical and surgical intervention units for all race groups supported the portability of the education bundle.

The present work extends previous initiatives designed and implemented to reduce disparities in health care delivery.[38–40] Strategies to identify and address inequalities in the quality of care that minority patients receive is emerging and one goal of Healthy People 2020.[41] Our overall goal was to improve the quality of care for all patients. However, non-differential improvement of doses accepted by patients was always a secondary goal of our project. Our approach to ensure health care equity was to target the subjective culture, comprising perceptions, attitudes, beliefs, and stereotypes surrounding the silent but debilitating and deadly nature of VTE. We engaged a representative group of local and national patient stakeholders to understand what they wanted to know about VTE and how they preferred to learn. The purpose was to develop education that targeted their preferences while improving their health literacy about VTE and appropriate prevention and their ability to make informed care decisions.[30]

Previous research has shown that racial and ethnic minorities tend to receive lower quality of care than non-minorities related to patient, process, and structural factors.[42–44] Consistent with best practices of patient-centered care delivery,[45] our study highlighted the relevance of leveraging health information technology as a quality improvement strategy to improve care through adherence to administered doses of pharmacologic VTE prophylaxis. Our approach also points to policies that support the advancement of risk-appropriate VTE prophylaxis through a risk assessment tool built into our EHR that follows best practice recommendations for VTE.[25] We concurrently attempted to break the gaps between prescription and administration practices by improving health literacy, which by its nature can be distributed equally across all race subpopulations.

Quality improvement efforts and initiatives have skyrocketed nationwide for well over a decade. While we used quality improvement to explore and eliminate disparities,[22] we did not find other groups who examined the differential effect of race on their study findings. Moving forward, we suggest that all quality improvement initiatives be rigorously examined to ensure there is no differential effect by race, and other demographics such as age, sex, gender, and ethnicity, as appropriate.[22] While some may say that this is a negative study, we still see benefit in objectively studying and publishing these types of data to ensure quality improvement efforts do not inadvertently worsen health disparities.

Our study has limitations. First, our analyses did not examine the effect of patient-provider race concordance. The difficulty in making definitive assertions about the role of race concordance for the missed doses due to other reasons points to the value of additional research. Second, our findings reflect a single medical institution and our patient demographics may not be representative of other medical facilities. Third, as we looked at smaller and smaller strata, we may have lost power to detect statistically significant differences. For example, in the strata by race for the outcome of refused doses, the point estimates for the effect sizes were similar to the overall numbers (in the vicinity of about a 40% to 50% improvement), yet the confidence intervals were wide and *p* values above 0.05. While we cannot convincingly claim statistical significance, we did find that the estimates were in the same direction and of similar magnitude. Fourth, unknown patient engagement interventions by physicians regarding VTE prevention may have impacted our results, but these likely would have been the same between pre- and post-periods and between floors.

Projections from the 2010 US Census estimate that racial and ethnic minority populations will substantially increase and eventually be the majority by 2050.[41,46] It is crucial to implement effective health education interventions that incorporate an understanding of the cultural values, perceptions, and attitudes of a diverse, multiethnic patient population and involve this audience as stakeholders in the processes from inception to implementation, placing greater emphasis on improving the quality of care and ensuring equity. Quality improvement efforts should consciously target all patient populations equally, regardless of race, ethnicity, sex, gender, or age.

## Supporting information

S1 Table Demographic characteristics of patient visits by intervention and control groups stratified by race. (DOCX)

S1 Trial protocol (PDF)

S1 Trend checklist TREND statement checklist. (PDF)

S1 Fig (TIF)
