# Supplementary material for: Using electronic health record system triggers to target delivery of a patient-centered intervention to improve venous thromboembolism prevention for hospitalized patients: Is there a differential effect by race?
Source: PLoS One. 2020 Jan 16;15(1):e0227339. doi: 10.1371/journal.pone.0227339 (PMC6964816; doi:10.1371/journal.pone.0227339)
Supplement: S1 Table — (DOCX) [file pone.0227339.s001.docx]

Supplement 1. Demographic Characteristics of Patient Visits by Intervention and Control Groups Stratified by Race

| **Race** | **Baseline Characteristics** | **Intervention** | | | | **Control** | | | | **p-value comparing pre intervention and pre control** | |
| --- | --- | --- | --- | --- | --- | --- | --- | --- | --- | --- | --- |
|  |  | **Pre period** | **Post period** | **p-value** | **Pre period** | | **Post period** | **p-value** |  | |  |
| **Black** | **Unique Visits** | 941 | 1308 |  | 2307 | | 3469 |  |  | |  |
|  | **Unique Patients** | 762 | 1039 |  | 1860 | | 2635 |  |  | |  |
|  | **Unique Nurses** | 100 | 114 |  | 268 | | 335 |  |  | |  |
|  | **Mean Age (SD), years^a^** | 51.8 (17.4) | 51.8 (18.1) | 0.99 | 54.3 (16.7) | | 53.2 (16.8) | 0.02 | <0.001 | |  |
|  | **Sex, n (%)^b^**  **Male**  **Female** | 449 (47.8%)  491 (52.2%) | 637 (48.7%)  671 (51.3%) | 0.66 | 1083 (46.9%)  1224 (53.1%) | | 1712 (49.4%)  1755(50.6%) | 0.07 | 0.67 | |  |
|  | **Floor Type, n (%)^b^**  **Surgery Floors**  **Medicine Floors** | 319 (33.9%)  622 (66.1%) | 482 (36.9%)  826 (63.1%) | 0.15 | 528 (22.9%)  1779 (77.1%) | | 907 (26.1%)  2562 (73.9%) | 0.005 | <0.001 | |  |
|  | **Median Number of Prescribed Doses per Patient visit (Q1, Q3)**  **Mean (SD)^c^** | 6 (3-11)  8.5 (8.6) | 6 (3-12)  9.72 (15.5) | 0.12 | 7 (3-13)  10.3 (10.9) | | 7 (3-14)  10.8 (12.5) | 0.52 | <0.001 | |  |
|  | **Median Length of Stay, days (Q1-Q3)**  **Mean (SD)^c^** | 3 (2-6)  5.2 (6.4) | 4 (2-7)  6.0 (9.3) | 0.06 | 4 (2-7)  6.0 (8.0) | | 4 (2-8)  6.5 (8.7) | 0.03 | <0.001 | |  |
| **White** | **Unique Visits** | 1,088 | 1,533 |  | 2,850 | | 4,432 |  |  | |  |
|  | **Unique Patients** | 922 | 1,279 |  | 2,428 | | 3,669 |  |  | |  |
|  | **Unique Nurses** | 121 | 131 |  | 409 | | 421 |  |  | |  |
|  | **Mean Age (SD), years^a^** | 56.1 (17.3) | 57.7 (16.6) | 0.02 | 58.5 (16.7) | | 58.3 (16.5) | 0.49 | <0.001 | |  |
|  | **Sex, n (%)^b^**  **Male**  **Female** | 526 (48.3%)  562 (51.7%) | 738 (89.1%)  795 (51.9%) | 0.92 | 1599 (56.1%)  1251 (43.9%) | | 2438 (55.0%)  1994 (45.0%) | 0.36 | <0.001 | |  |
|  | **Floor Type, n (%)^b^**  **Surgery Floors**  **Medicine Floors** | 717 (65.9%)  371 (34.1%) | 1012 (66.0%)  521 (34.0%) | 0.95 | 1732 (60.8%)  1118 (39.2%) | | 2736 (61.7%)  1696 (38.3%) | 0.41 | 0.003 | |  |
|  | **Median Number of Prescribed Doses per Patient visit (Q1, Q3)**  **Mean (SD)^c^** | 7 (3-14)  10.5 (11.3) | 8 (4-14)  11.2 (11.9) | 0.12 | 8 (4-15)  12.0 (14.1) | | 8 (4-15)  11.7 (12.1) | 0.21 | <0.001 | |  |
|  | **Median Length of Stay, days (Q1-Q3)**  **Mean (SD)^c^** | 4 (2-7)  6.0 (5.8) | 5 (3-8)  6.5 (6.7) | 0.09 | 5 (2-8)  7.3 (9.4) | | 5 (3-8)  7.5 (10.4) | 0.10 | 0.004 | |  |
| **Other** | **Unique Visits** | 193 | 270 |  | 500 | | 761 |  |  | |  |
|  | **Unique Patients** | 170 | 221 |  | 431 | | 648 |  |  | |  |
|  | **Unique Nurses** | 27 | 29 |  | 67 | | 79 |  |  | |  |
|  | **Mean Age (SD), years^a^** | 51.7 (16.4) | 50.6 (17.6) | 0.50 | 53.3 (16.7) | | 53.5 (17.6) | 0.89 | 0.26 | |  |
|  | **Sex, n (%)^b^**  **Male**  **Female** | 93 (48.2%)  100 (51.8%) | 149 (55.2%)  121 (44.8%) | 0.14 | 288 (57.6%)  212 (42.4%) | | 435 (57.2%)  328(42.8%) | 0.90 | 0.03 | |  |
|  | **Floor Type, n (%)^b^**  **Surgery Floors**  **Medicine Floors** | 119 (61.7%)  74 (38.3%) | 153 (56.7%)  117 (43.3%) | 0.28 | 270 (54.0%)  230 (46.0%) | | 433 (56.9%)  328 (43.1%) | 0.31 | 0.07 | |  |
|  | **Median Number of Prescribed Doses per Patient visit (Q1, Q3)**  **Mean (SD)^c^** | 6 (3-14)  11.3 (14.0) | 6.5 (3-13)  10.4 (11.0) | 0.93 | 8 (4-14)  10.8 (10.9) | | 8 (4-15)  12.7 (17.9) | 0.12 | 0.55 | |  |
|  | **Median Length of Stay, days (Q1-Q3)**  **Mean (SD)^c^** | 4 (2-8)  7.6 (11.4) | 4 (2-7)  6.3 (7.9) | 0.85 | 5 (2-8)  7.2 (8.8) | | 5 (2-9)  8.3 (12.4) | 0.18 | 0.24 | |  |

^a^ The p values were calculated using two-sample t-tests with equal variances.

^b^ The p values were calculated using chi-square tests.

^c^ The p values were calculated using Wilcoxon rank-sum tests.
