# Supplementary figures and images for: Using electronic health record system triggers to target delivery of a patient-centered intervention to improve venous thromboembolism prevention for hospitalized patients: Is there a differential effect by race?
Source: PLoS One. 2020 Jan 16;15(1):e0227339. doi: 10.1371/journal.pone.0227339 (PMC6964816; doi:10.1371/journal.pone.0227339)

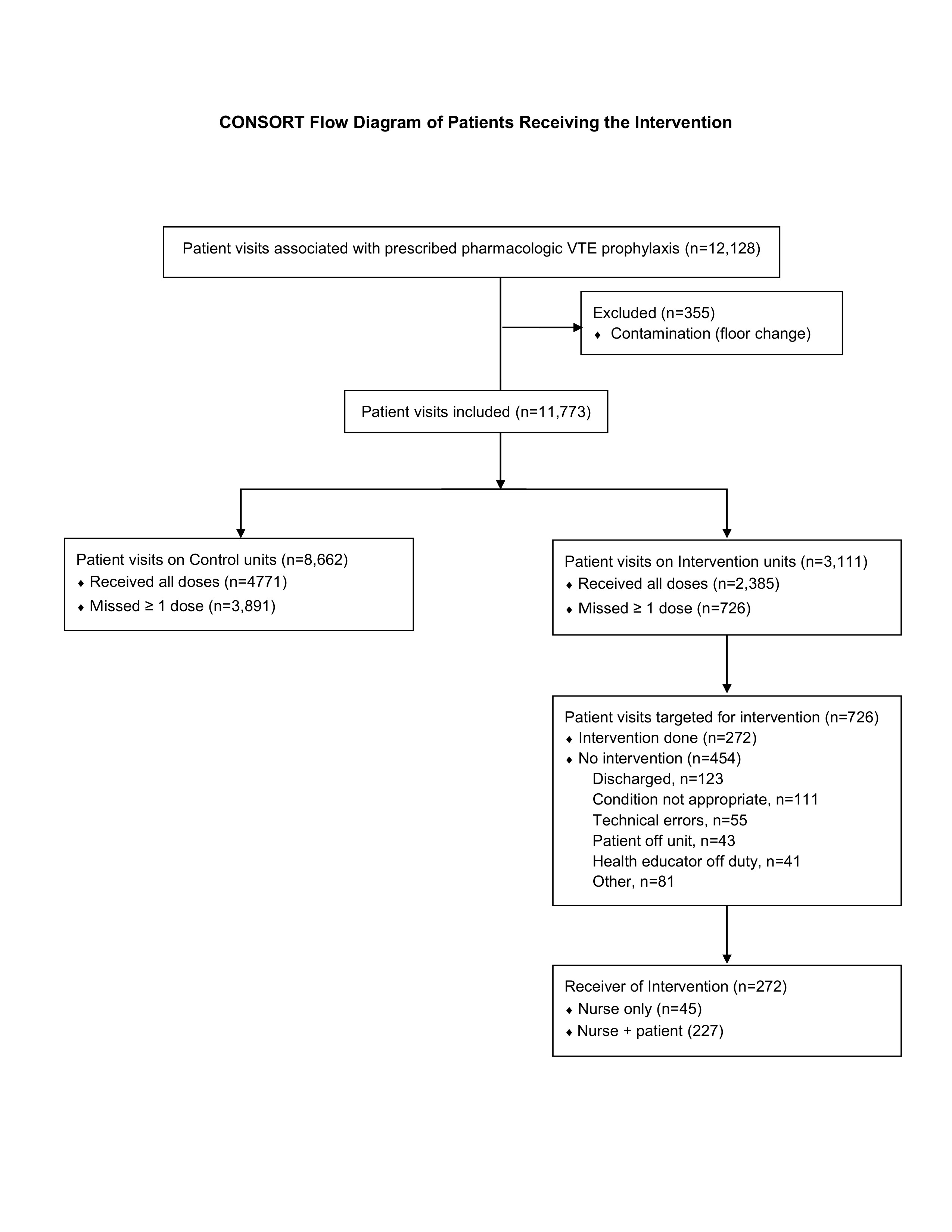

Supplement: S1 Fig — (TIF) [file pone.0227339.s004.tif]
